# Citrullinemia type I is associated with a novel splicing variant, c.773 + 4A > C, in *ASS1*: a case report and literature review

**DOI:** 10.1186/s12881-019-0836-5

**Published:** 2019-06-17

**Authors:** Yiming Lin, Hongzhi Gao, Bin Lu, Shuang Zhou, Tianwen Zheng, Weihua Lin, Lin Zhu, Mengyi Jiang, Qingliu Fu

**Affiliations:** 1 Neonatal Disease Screening Center, Quanzhou Maternity and Children’s Hospital, 700 Fengze Street, Quanzhou, 362000 Fujian Province China; 2 Department of Central Laboratory, 2nd Affiliated Hospital of Fujian Medical University, Quanzhou, 362000 Fujian Province China; 3 Genuine Diagnostics Company Limited, 859 Shixiang West Road, Hangzhou, 310007 Zhejiang Province China; 4 Department of Pediatrics, Quanzhou Maternity and Children’s Hospital, 700 Fengze Street, Quanzhou, 362000 Fujian Province China

**Keywords:** Citrullinemia type I, *ASS1*, Novel variant, Mutation spectrum

## Abstract

**Background:**

Citrullinemia type I (CTLN1) is a rare autosomal recessive disorder of the urea cycle caused by a deficiency in the argininosuccinate synthetase (ASS1) enzyme due to mutations in the *ASS1* gene. Only a few Chinese patients with CTLN1 have been reported, and *ASS1* gene mutations have been identified sporadically in China.

**Case presentation:**

A Chinese family with one member affected with mild CTLN1 was enrolled. Targeted exome sequencing was performed on the proband, and Sanger sequencing was used to validate the detected mutation. We also reviewed the genetic and clinical characteristics of CTLN1 in Chinese patients that have been published to date. Newborn screening showed remarkably increased concentrations of citrulline with elevated ratios of citrulline/arginine and citrulline/phenylalanine, and the patient presented with a speech delay at age three. The urinary organic acid profiles were normal. A novel homozygous splicing variant c.773 + 4A > C in the *ASS1* gene was identified in the proband, and it was predicted to affect splicing by in silico analysis. To date, only nine Chinese patients with CTLN1 have been reported, with a total of 15 *ASS1* mutations identified and no high frequency or hot spot mutations found; the mutation spectrum of Chinese patients with CTLN1 was heterogeneous.

**Conclusions:**

We described a mild Chinese CTLN1 case with a novel homozygous splicing variant c.773 + 4A > C and reviewed previous genotypes and phenotypes in Chinese patients with CTLN1. Thus, our findings contribute to understanding the molecular genetic background and clinical phenotype of CTLN1 in this population.

**Electronic supplementary material:**

The online version of this article (10.1186/s12881-019-0836-5) contains supplementary material, which is available to authorized users.

## Background

Citrullinemia type I (CTLN1, MIM# 215700) is a rare autosomal recessive disorder of the urea cycle caused by a deficiency of the argininosuccinate synthetase (ASS, EC 6.3.4.5) enzyme due to mutations in the *ASS1* gene [1]. CTLN1 encompasses a spectrum of varying clinical phenotypes. Patients that present with fatal neonatal hyperammonemia are said to have classical citrullinemia, patients with late onset and/or mild symptoms are said to have mild citrullinemia, and a considerable number of asymptomatic individuals detected by expanded newborn screening (NBS) have only a biochemical phenotype [2, 3].

Biochemically, CTLN1 with elevated citrulline concentrations can be detected by NBS. Nonetheless, increased levels of citrulline can also be found in other inherited metabolic disorders such as citrullinemia type II, argininosuccinate lyase deficiency, and pyruvate carboxylase deficiency [4, 5]. Therefore, definitive diagnosis of CTLN1 mainly relies on an ASS enzyme assay and identification of *ASS1* gene mutations. However, determination of enzyme activity in liver tissue requires an invasive procedure, and direct measurement of ASS activity or indirect measurement using a C^14^ incorporation assay in fibroblasts have not yet been evaluated in patients with mild CTLN1 [6]. Therefore, molecular genetic testing is paramount, not only for clinical diagnosis but also for future prenatal testing and family member screening [7].

The *ASS1* gene is located on chromosome 9q34.1 and contains 16 exons, with the translation start codon in exon 3, encoding 412 amino acids [2, 8]. To date, At least 137 mutations that cause CTLN1 have been reported in the *ASS1* gene [9]. However, only a few Chinese patients with CTLN1 have been reported, and *ASS1* gene mutations have been identified only sporadically in China [10–17]. In this study, we present biochemical, clinical, and genetic characteristics of a new Chinese patient with CTLN1. In addition, we reviewed previous genotypes and phenotypes of Chinese patients with CTLN1, to help better understand the genetic background of this disease in the Chinese population.

## Case presentation

### Case report

This study was approved by the Ethical Committee of Quanzhou Maternity and Children’s Hospital. Written informed consent was obtained from the parents of the patient, who agreed to join this study, with the intent of using the resulting medical data for scientific research and publication. The proband was born at a gestational age of 38 weeks and 1 day via caesarean section; his weight at birth was 3700 g. He was the third born child of consanguineous parents of Chinese descent. There was no significant family history of inherited metabolic diseases. NBS via ACQUITY TQD tandem mass spectrometry (MS/MS) (Waters, Milford, MA, USA) analysis on dried blood spots (DBS) was performed on the proband after birth. The initial NBS results showed an elevated citrulline concentration with increased ratios of citrulline/arginine and citrulline/phenylalanine. The hypercitrullinemia and increased citrulline/phenylalanine ratios persisted, and the concentrations of citrulline fluctuated between 71.82–120.99 μmol/L during follow up, while the citrulline/arginine ratios were persistent within the reference range (Table 1). Subsequently, urinary organic acid analysis by gas chromatography-mass spectrometry (7890B/5977A, Agilent Technologies, Santa Clara, CA, USA) and auxiliary biochemical tests were carried out. Increased levels of orotic acid were not observed in urinary organic acid analysis. The blood ammonia levels were slightly elevated at 1 month and 11 months, which may have been transitional, and returned to the normal reference range later. The patient exhibited normal growth and development during follow up, but a speech delay was noted at 3 years of age.

**Table 1:** Detection results of MS/MS and biochemical testing in the patient

Testing time	MS/MS analysis in dried blood spots				Biochemical testing			
	Citrulline (5.5–30 μmol/L)	Citrulline/Arginine (0.35–15)	Citrulline/Phenylalanine (0.12–0.83)	Arginine (1–50 μmol/L)	Blood ammonia (10–47 μmol/L)	Total bilirubin (5.1–19 μmol/L)	Direct bilirubin (0–6.8 μmol/L)	AFP (ng/ml)
2015.9.21^a^	90.05	46.18	1.17	1.95				
2015.10.9	89.87	6.58	2.26	13.65				
2015.10.19	88.14	4.72	2.82	18.69	70	142	15.6	6508.14
2016.8.26	118.24	3.9	2.56	30.3	49	4.2	0.9	2.37
2016.10.10	87.66	4.8	1.9	18.25	33			
2016.11.8	81.25	3.52	2.25	23.05				
2017.8.4	71.82	5.19	1.37	13.85	29	6.2	1.6	<1.3
2018.9.7	120.99	3.4	1.45	35.63	45	3.2	2.1	

^a^Newborn screening results

### Genetic analysis

Genomic DNA was extracted from whole blood of the proband and his family members using Qiagen Blood DNA mini kit (Qiagen®, Hilden, Germany). The DNA of the proband was used for NGS. Targeted enrichment of target region sequences was performed by multiple probe hybridization using metabolic abnormality of common amino acids capture oligo, which was designed by Genuine Diagnostics (Zhejiang, China), and included 40 genes (*ABCD4, ACSF3, ARG1, AMT, ASS1, ASL, BCAT1, BCAT2, BCKDHA, BCKDHB, CBS, CPS1, CTH, D2HGDH, ETHE1, GCH1, GCSH, GLDC, GPHN, GRHPR, HGD, HPD, L2HGDH, LMBRD1, MOCS1, MOCS2, MTR, OAT, OGDH, OPA3, PAH, PCBD1, PRODH, PTS, QDPR, SERAC1, SLC25A13, SLC25A15, SPR, and SUOX*). The sequencing libraries were quantified using the Illumina DNA Standads and Primer Premix Kit (Kapa Biosystems, Boston, MA, USA), and then massively parallel sequenced using the Illumina HiSeq 2500 platform (Illumina, San Diego, CA, USA). After sequencing and filtering out low-quality reads, high-quality reads were compared to the human genome reference sequence (GRCh37.p13, hg19). The quality control data are listed in Additional file 1: Table S1. Variants were called using the GATK software. Next, candidate variants were further confirmed by Sanger sequencing. Sequencing was performed on ABI 3500xL (Applied Biosystems, Foster City, CA, USA), and the results were analysed using DNASTAR software. The primers used for polymerase chain reaction (PCR) and Sanger sequencing are listed in Additional file 2: Table S2.

The identified variants were annotated to public databases, such as the Human Gene Mutation Database (HGMD, http://www.hgmd.cf.ac.uk/ac/index.php), ClinVar (https://www.ncbi.nlm.nih.gov/clinvar/), the Leiden Open Variation Database (LOVD, http://www.lovd.nl/3.0/home), dbSNP (https://www.ncbi.nlm.nih.gov/projects/SNP/), the 1000 Genome Project (http://www.1000genomes.org/), and the ExAC consortium (http://exac.broadinstitute.org/). In addition, the variant was further assessed for possible pathogenicity using HSF (http://www.umd.be/HSF3/), MutationTaster (http://www.mutationtaster.org/), and regSNP-intron (http://clark.compbio.iupui.edu/regsnp_intron_web/). To exclude any polymorphisms, 100 healthy controls underwent Sanger sequencing of the *ASS1* exon 11. Pathogenicity analysis of the variant was performed to comply with the American College of Medical Genetics and Genomics (ACMG) guidelines [18].

### Mutation analysis

**Fig. 1:**
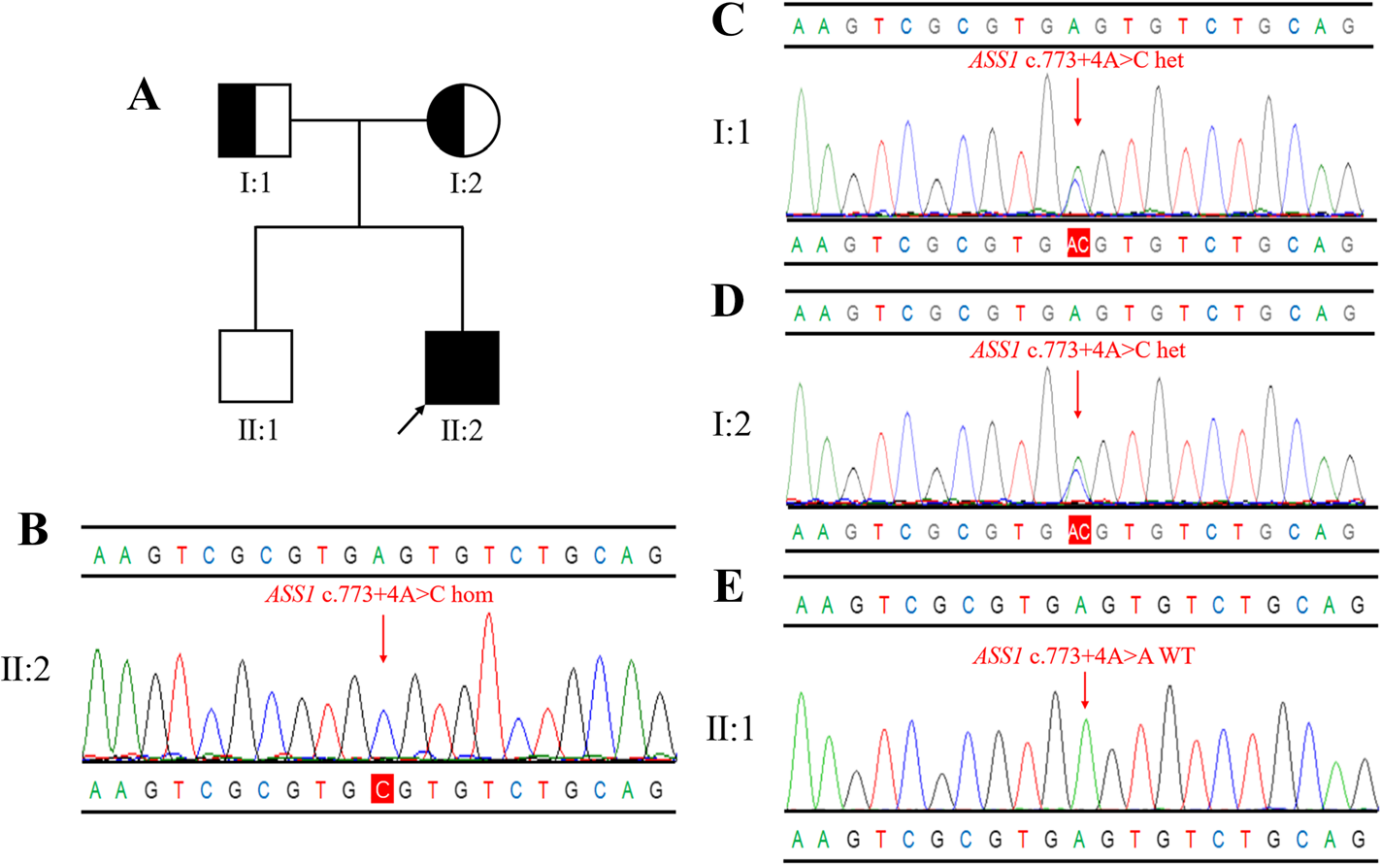
**a**: Pedigree of the family. The filled black symbols represent the affected members and the arrow denotes the proband. **b**-**e**: Sequence analysis of the *ASS1* gene independently identified the c.773 + 4A > C variant in the proband (**b**), his father (**c**), his mother (**d**), and his old brother (**e**)

We identified a homozygous *ASS1* gene variant c.773 + 4A > C in the proband, which was inherited from the parents. In addition, no *ASS1* mutations were detected in the proband’s older brother via family genetic screening (Fig. 1). The c.773 + 4A > C variant is located in intron 11 of the *ASS1* gene, with the 5th nucleotide changed from adenine to cytosine. This variant has not been previously reported in the literature and was not detected in 100 healthy controls. It was found only in a heterozygous state in the East Asian population with an allele frequency of 1.387e-03 in the ExAC database, and the allele frequency in the overall population was 9.892e-05, indicating that individuals in the population with this heterozygous variation were just carriers. It was absent from the dbSNP and 1000 Genomes databases, and was also absent from disease databases such as HGMD, ClinVar, and LOVD. Furthermore, in silico analysis by HSF, MutationTaster, and regSNP-intron all suggested that the variant most likely affects splicing (Table 2). According to the ACMG guidelines, the c.773 + 4A > C variant was classified as variant of uncertain clinical significance.

**Table 2:** The effect of c.773 + 4A > C on protein function by in silico analysis

Software	Score	Predicted signal
HSF^a^	Site broken (−42.85)	Alteration of the WT donor site, most probably affect splicing
MutationTaster^b^	1	Disease causing
regSNP-intron^c^	0.822174865	Disease causing

^a^HSF: www.umd.be/HSF/. The score less than 0 is site broken

^b^MutationTaster: www.mutationtaster.org. The score is between 0 and 1, it is more likely to be disease causing with the score closer to 1

^c^regSNP-intron: http://clark.compbio.iupui.edu/regsnp_intron_web/. The score is between 0 and 1, it is more likely to be disease causing with the score closer to 1

In a review of Chinese CTLN1 cases published to date, we found that only ten Chinese patients with CTLN1 received genetic testing. The mutations in the *ASS1* gene from our study and from previously reported Chinese patients are summarized in Table 3. In total, 15 *ASS1* mutations have been identified in Chinese patients to date. Among them, 11 are missense mutations, three are splice mutations, and one is a deletion mutation. 50% of the mutant alleles are clustered in exons 7, 13, and 14. Four mutations (p.Arg127Gln, p.Arg265Cys, p.Gly324Ser, and p.Gly390Arg) proved to be disease-causing. Four patients had homozygous or compound heterozygous splicing/frameshift mutations, resulting in neonatal onsets and with poor outcomes, and thus were classified as having neonatal CTLN1. Three patients developed symptoms after the neonatal period with moderate outcomes and were classified as having late-onset CTLN1. The other three patients without obvious clinical symptoms and with good prognosis were classified as having a mild form of CTLN1.

**Table 3:** Clinical presentations, biochemical, and genetic investigations of ten Chinese patients with citrullinemia type 1

Patient no.	Gender	Age of onset	Clinical presentation	Citrulline levels (μmol/L)^a^	Blood ammonia (μmol/L)^b^	Mutaion 1			Mutaion 2			Outcome	Ref.
						Location	c.DNA^c^	Protein	Location	c.DNA	Protien		
1	Male	3 y	Mild form	90.05	70	Intron 11	**c.773 + 4A > C**		Intron 11	**c.773 + 4A > C**		Well	This study
2	n.a.	2 d	Neonatal form	487.69	286	Exon 6	c.380G > A	p.Arg127Gln	Exon 6	c.380G > A	p.Arg127Gln	Died	[12]
3	Male	n.p.	Mild form	961.42	91	Intron 4	c.174 + 1G > A		Exon 7	c.422 T > C	p.Val141Gly	Well	[13]
4	Female	4 d	Neonatal form	1085.41	231	Intron 11	c.773 + 1G > A		Exon 12	c.793C > T	p.Arg265Cys	Moderate	[13]
5	Female	3 m	Late-onset form	n.a.	311	Exon 7	c.431C > G	p.Pro144Arg	Exon 14	c.1087C > T	p.Arg363Trp	Moderate	[17]
6	Male	n.p.	Mild form	111.21	17	Exon 3	c.53C > T	p.Ser18Leu	Exon 15	c.1168G > A	p.Gly390Arg	Well	[16]
7	Female	1 y, 3 m	Late-onset form	928.77	160	Exon 13	c.847G > A	p.Glu283Lys	Exon 14	c.1009 T > C	p.Cys337Arg	Moderate	[10]
8	Female	1 y, 5 m	Late-onset form	653	126	Exon 5	c.236C > T	p.Ser79Phe	Exon 7	c.431C > G	p.Pro144Arg	n.a.	[14]
9	Female	2 d	Neonatal form	1577.7	670	Exon 13	c.951delT	p.F317LfsX375	Exon 14	c.1087C > T	p.Arg363Trp	Died	[11]
10	Male	2 d	Neonatal form	2513.5	n.a.	Exon 13	c.970G > A	p.Gly324Ser	Exon 13	c.970G > A	p.Gly324Ser	n.a.	[15]

^a^Reference range: 5.5–30 μmol/L; ^b^ Reference range: 10–47 μmol/L; ^c^ novel mutations are in bold character

*n.p*. not present, *n.a.* not available, *d* day, *y* year

## Discussion and conclusions

In this study, we described a Chinese family with one child having a mild form of CTLN1. The patient had an elevated citrulline level, which was detected by MS-based NBS. No abnormalities were found in urinary organic acid analysis. The patient had normal growth and development during follow up, and the main clinical manifestation was a speech delay. A homozygous *ASS1* gene variant c.773 + 4A > C was identified in the patient. This variant has not been previously reported in the literature, and was predicted through bioinformatics analysis to cause a broken WT donor site and to affect splicing. Furthermore, it is likely to truncate the monomers, impairing the synthetase-binding domain. Similar variants leading to protein truncation have been reported previously [13, 19, 20]. Therefore, we believe that the variant c.773 + 4A > C is associated with the pathogenesis of CTLN1. However, further functional studies are needed to validate the pathogenicity of this variant.

After combining our findings with those from previously reported Chinese patients with CTLN1, we performed mutation spectrum analysis. The results showed that the mutation spectrum of Chinese patients with CTLN1 was heterogeneous, with no high frequency or hot spot mutations. In comparison, many mutations have been documented at high frequencies in various other populations and the mutation spectra differ among different ethnic groups. p.Gly390Arg is by far the most common mutation and is widely distributed around the world [9]. It has been proposed that a CpG dinucleotide in the coding region could be the cause of recurring mutations in this region [21], and therefore the recurrent nature of this variant could be explained by its location in a CpG dinucleotide. For instance, the allelic frequencies of p.Gly390Arg in Indian and Turkish patients are 42.7 and 50%, respectively [22, 23]. Likewise, 17.3% of German patients carry at least one p.Gly390Arg allele. p.Gly390Arg is also regarded as a recurrent mutation in a limited geographic area of Argentina [24]. Similarly, the p.Val263Met variant seems to be common in the Pacific Island population [25]. The most frequent mutations in Korean patients are c.421-2A > G, p.Gly324Ser, and c.1128-6_1188dup67, and in Japanese patients the predominant mutations are c.421-2A > G, p. Arg265His, and p.Arg304Trp [26–30]. However, it is surprising that the particularly frequent mutation c.421-2A > G, reported in Korean and Japanese patients, has not yet been detected in Chinese patients.

To date, some mutations have been elucidated with clear genotype-phenotype correlations [9], while most Chinese patients were in a compound heterozygous state, rendering it more difficult to investigate the relationship between genotype and phenotype. Though p.Arg127Gln was proven to be inactive in previous studies, in the neonatal CTLN1 group, a patient (no.2) in our study homozygous for p.Arg127Gln died shortly after birth, confirming previous reports and highlighting the severity of this mutation [31]. Previous enzyme studies revealed that both p.Arg265Cys and p.Gly324Ser yielded < 2% of ASS wild-type activity, and both are known to be associated with a severe phenotype [32]. Supporting these findings, a patient (no.4) who was compound heterozygous for p.Arg265Cys with a splicing mutation presented with early onset neonatal citrullinmia; of note is that an older sibling in this family progressed to severe encephalopathy and died 4 days after birth. A patient (no.10) homozygous for p.Gly324Ser presented with acute hyperammonemia and encephalopathy, again confirming previous studies. p.Arg363Trp was reported to be associated with neonatal CTLN1; consistent with this, a patient (no.9) with p.Arg363Trp in combination with a frameshift mutation died shortly after birth [9]. Regarding mild/late-onset form CTLN1, a patient (no.1) homozygous for c.773 + 4A > C presented no clinical symptoms until 3 years of age, indicating that this variant may be related to mild symptoms. The remaining patients are all compound heterozygotes, and it is likely that the mutations p.Ser18Leu, p.Val141Gly, p.Pro144Arg, and p.Cys337Arg may allow for some residual ASS function, because the second allelic mutations in these patients are known to drastically impair ASS activity [9, 13, 32].

In summary, we described one mild Chinese CTLN1 case with a novel splicing variant c.773 + 4A > C. We also reviewed previous genotypes and phenotypes of Chinese patients with CTLN1, hereby adding to our understanding of the molecular genetic background and clinical phenotype of CTLN1 in this population. The mutation spectrum of Chinese patients with CTLN1 was heterogeneous. More functional research is needed to elucidate the genotype-phenotype correlation in patients with CTLN1.

## Additional files

Additional file 1: **Table S1.** Summary of targeted gene sequencing data in the proband. (DOCX 13 kb)

Additional file 2: **Table S2.** Primers used for PCR and Sanger sequencing of exon 11 of ASS1. (DOCX 14 kb)

## Data Availability

The datasets used and/or analysed during the current study are available from the corresponding author upon reasonable request.

## Abbreviations

ACMG: American College of Medical Genetics Association of Clinical Genetics
CTN1: Citrullinemia type I
MAF: Minor allele frequency
MS/MS: Tandem mass spectrometry
NBS: Newborn screening
NGS: Next-generation sequencing
PCR: Polymerase chain reaction
