# Supplementary material for: Citrullinemia type I is associated with a novel splicing variant, c.773 + 4A > C, in ASS1: a case report and literature review
Source: BMC Med Genet. 2019 Jun 17;20:110. doi: 10.1186/s12881-019-0836-5 (PMC6580464; doi:10.1186/s12881-019-0836-5)
Supplement: Supplementary file 1 — Table S1. Summary of targeted gene sequencing data in the proband. (DOCX 13 kb) [file 12881_2019_836_MOESM1_ESM.docx]

**Additional file 1: Table S1**.Summary of targeted gene sequencing data in the proband.

| Raw reads | 1165469 |
| --- | --- |
| Unique mapping reads ratio | 99.42% |
| Mean read depth on target | 153.54 |
| Fraction of target covered with at least 1X | 100% |
| Fraction of target covered with at least 20X | 96.92% |
| Fraction of target covered with at least 50X | 93.28% |
| Fraction of target covered with at least 100X | 90.26% |
