# Supplementary material for: Citrullinemia type I is associated with a novel splicing variant, c.773 + 4A > C, in ASS1: a case report and literature review
Source: BMC Med Genet. 2019 Jun 17;20:110. doi: 10.1186/s12881-019-0836-5 (PMC6580464; doi:10.1186/s12881-019-0836-5)
Supplement: Supplementary file 2 — Table S2. Primers used for PCR and Sanger sequencing of exon 11 of ASS1. (DOCX 14 kb) [file 12881_2019_836_MOESM2_ESM.docx]

**Additional file 2: Table S2** Primers used for PCR and Sanger sequencing of *ASS1* exon 11.

| Primer | Name | Sequence (5′→3′) |
| --- | --- | --- |
| PCR | *ASS1*-e11F | TGTAAAACGACGGCCAGTTTTCTCGCCCCTTCTCTC |
|  | *ASS1*-e11R | CAGGAAACAGCTATGACCAGCCTCAGCCACAACCAT |
| Sanger Sequencing | M13F | TGTAAAACGACGGCCAGT |
|  | M13R | CAGGAAACAGCTATGACC |
